# Supplementary material for: Global population genomic signature of Spodoptera frugiperda (fall armyworm) supports complex introduction events across the Old World
Source: Commun Biol. 2022 Apr 7;5:297. doi: 10.1038/s42003-022-03230-1 (PMC8989990; doi:10.1038/s42003-022-03230-1)
Supplement: Supplementary file 2 — Supplementary Information [file 42003_2022_3230_MOESM2_ESM.pdf]

Global population genomic signature of *Spodoptera frugiperda* (fall armyworm) supports complex introduction events across the Old World

Wee Tek Tay[1], Rahul V. Rane[1], Amanda Padovan[1], Tom K. Walsh[1], Samia Elfekih[2], Sharon Downes[3], Kiwong Nam[4], Emmanuelle d'Alençon[4], Jianpeng Zhang[5], Yidong Wu[5], Nicolas Nègre[4], Daniele Kunz[6], Darren J. Kriticos[1], Cecilia Czepak[7], Michael H. Otim[8], Karl H.J. Gordon[1].

1. CSIRO Black Mountain Laboratories, Clunies Ross Street, Canberra, ACT 2602, Australia
2. CSIRO Australian Centre for Disease Preparedness, Geelong, Vic, Australia
3. CSIRO FD McMaster Laboratories, New England Highway, Armidale NSW2350, Australia
4. DGIMI, Université Montpellier, INRAE, Montpellier, France
5. College of Plant Protection Nanjing Agricultural University, Nanjing, China
6. Gordon Institute, University of Cambridge, Cambridge CB2 1QN, UK
7. Universidade Federal de Goiás, Escola de Agronomia, Goiânia, GO, Brazil
8. National Crops Resources Research Institute, Namulonge, Kampala, Uganda

(a) Native and invasive *Spodoptera frugiperda* admixture analysis

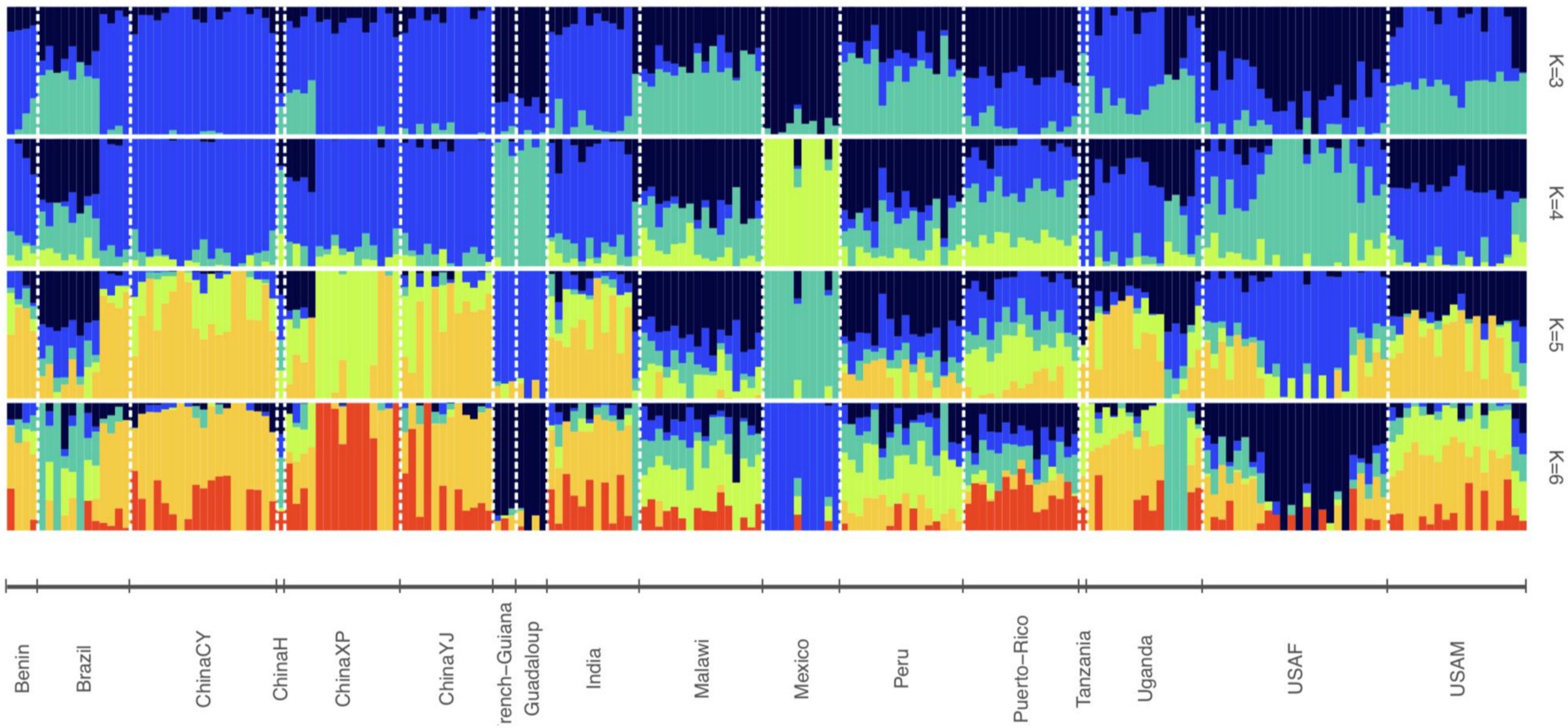

Supplementary Figure 1: (**a**) Admixture analysis based on 870 unlinked and neutral genome-wide SNPs for FAW populations from both native (North, Central, South Americas) and invasive (Africa, Asia) ranges. The Mexican population represents the only New World native FAW population with most individuals having non-admixed genome patterns, and where all individuals also have the corn mitogenome haplotype.

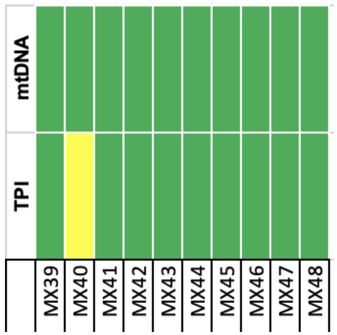

(b) Mitochondrial DNA genome and *Tpi* gene characterisation of Mexican FAW, showing one individual also have the rice *Tpi* signature (yellow colour cell). Corn mitochondrial genome and *Tpi* gene are in green colour.

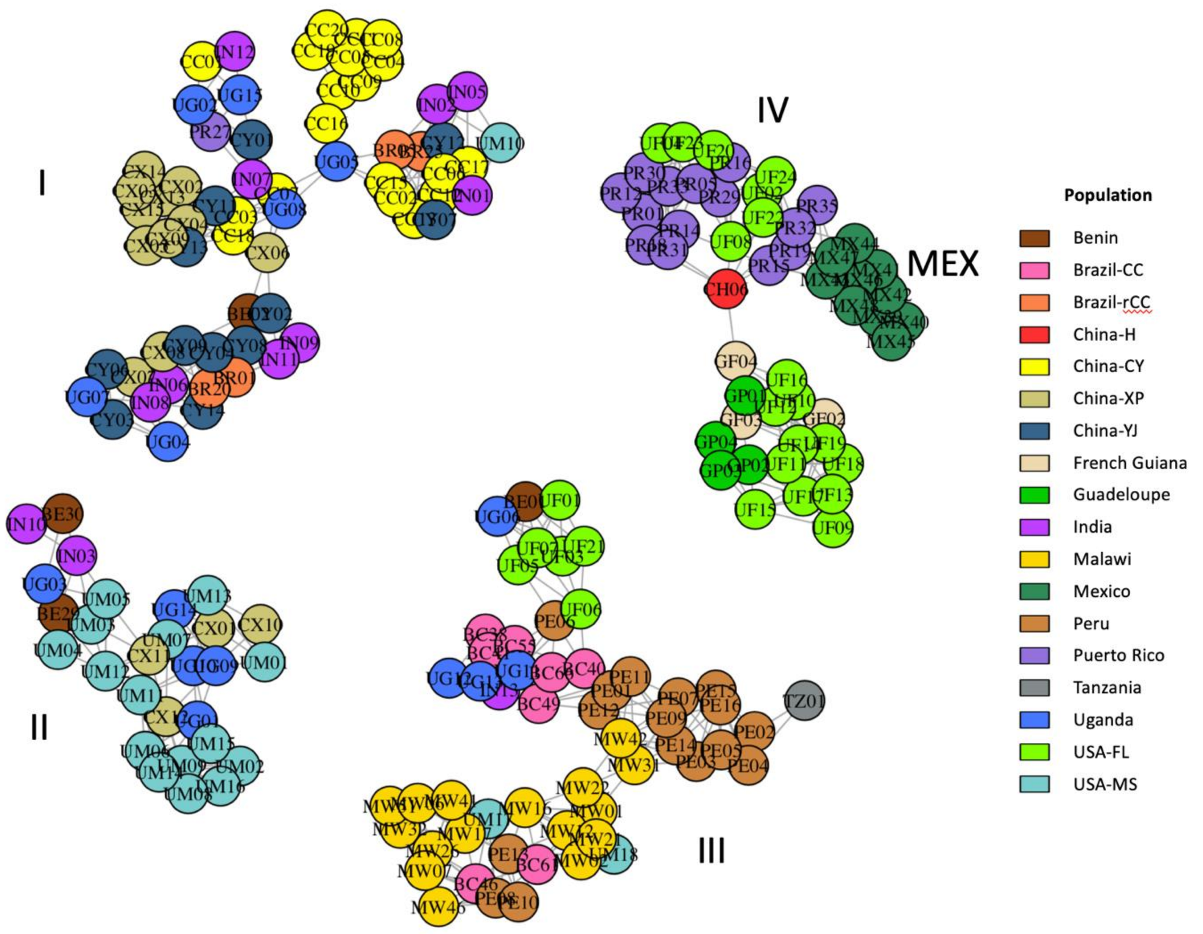

Supplementary Figure 2: Maximum Likelihood (ML) network showing individuals belonging to the populations as given in the legend. The network was drawn using the plotAdmixture function in the R package NetView [1,2], and is based on a ML distance matrix calculated from the IQ-Tree shown in Fig. 3. using the R package ape [3]. The four major clusters, I – IV, correspond to those shown in the tree. Individuals are identified by country codes as follows: China XP (CX), China YJ (CY), China CY (CC), China CH06 (CH), India (IN), Uganda (UG), Tanzania (TZ), Malawi (MW), Benin (BE), Brazil CC (BC), Brazil rCC (BR), Peru (PE), French Guiana (GF), Mexico (MX), Guadeloupe (GP), Puerto Rico (PR), USA-Florida (UF), and USA-Mississippi (UM). See Supplementary Data 1 for complete information about the individuals.

Supplementary References

1 Neuditschko, M., Khatkar, M. S. & Raadsma, H. W. NetView: a high-definition network-visualization approach to detect fine-scale population structures from genome-wide patterns of variation. *PLoS One* **7**, e48375, doi:10.1371/journal.pone.0048375 (2012).

2 Steinig, E. J., Neuditschko, M., Khatkar, M. S., Raadsma, H. W. & Zenger, K. R. netview p: a network visualization tool to unravel complex population structure using genome-wide SNPs. *Mol Ecol Resour* **16**, 216-227, doi:10.1111/1755-0998.12442 (2016).

3 Paradis, E. & Schliep, K. ape 5.0: an environment for modern phylogenetics and evolutionary analyses in R. *Bioinformatics* **35**, 526-528, doi:10.1093/bioinformatics/bty633 (2019).

4 Subramanian S. The effect of sample size on population genomic analyses - implications for the tests of neutrality. BMC Genomics 17, 123 (2016).
