# Supplementary material for: Global population genomic signature of Spodoptera frugiperda (fall armyworm) supports complex introduction events across the Old World
Source: Commun Biol. 2022 Apr 7;5:297. doi: 10.1038/s42003-022-03230-1 (PMC8989990; doi:10.1038/s42003-022-03230-1)
Supplement: Supplementary file 6 — Reporting Summary [file 42003_2022_3230_MOESM6_ESM.pdf]

nature research

COMMSBIO-20-2645-T
Corresponding author(s): TAY, WEE TEK
Last updated by author(s): Feb 2, 2022

Reporting Summary

Nature Research wishes to improve the reproducibility of the work that we publish. This form provides structure for consistency and transparency in reporting. For further information on Nature Research policies, see our Editorial Policies and the Editorial Policy Checklist.

Statistics

For all statistical analyses, confirm that the following items are present in the figure legend, table legend, main text, or Methods section.

n/a	Confirmed	
☐	☒	The exact sample size (*n*) for each experimental group/condition, given as a discrete number and unit of measurement
☐	☒	A statement on whether measurements were taken from distinct samples or whether the same sample was measured repeatedly
☒	☐	The statistical test(s) used AND whether they are one- or two-sided<br>*Only common tests should be described solely by name; describe more complex techniques in the Methods section.*
☒	☐	A description of all covariates tested
☒	☐	A description of any assumptions or corrections, such as tests of normality and adjustment for multiple comparisons
☒	☐	A full description of the statistical parameters including central tendency (e.g. means) or other basic estimates (e.g. regression coefficient) AND variation (e.g. standard deviation) or associated estimates of uncertainty (e.g. confidence intervals)
☒	☐	For null hypothesis testing, the test statistic (e.g. *F*, *t*, *r*) with confidence intervals, effect sizes, degrees of freedom and *P* value noted<br>*Give P values as exact values whenever suitable.*
☒	☐	For Bayesian analysis, information on the choice of priors and Markov chain Monte Carlo settings
☒	☐	For hierarchical and complex designs, identification of the appropriate level for tests and full reporting of outcomes
☒	☐	Estimates of effect sizes (e.g. Cohen's *d*, Pearson's *r*), indicating how they were calculated

Our web collection on statistics for biologists contains articles on many of the points above.

Software and code

Policy information about availability of computer code

Data collection	All programme codes used in this study have been cited. No custom program codes were used in this study
Data analysis	Geneious v11.1.5<br>MITOS<br>Trimmomatic (v0.39)<br>SAMTOOLS (v1.10)<br>BWA-MEM (v2.1)<br>BBMap (v38.81)<br>BCFtools (v1.10)<br>Plink2.0<br>PLINK v1.9<br>IQ-Tree<br>Dendroscope version 3.5.7<br>VCFtools<br>PopGenome package in R<br>Genepop (v4.7.5)<br>Admixture v1.3.0<br>NetView R package<br>'ape' R package<br>diveRsity R package<br>Gephi

For manuscripts utilizing custom algorithms or software that are central to the research but not yet described in published literature, software must be made available to editors and reviewers. We strongly encourage code deposition in a community repository (e.g. GitHub). See the Nature Research guidelines for submitting code & software for further information.

Data

Policy information about availability of data

All manuscripts must include a data availability statement. This statement should provide the following information, where applicable:

- Accession codes, unique identifiers, or web links for publicly available datasets
- A list of figures that have associated raw data
- A description of any restrictions on data availability

All assembled mitochondrial genomes have been submitted to GenBank (accession numbers MT897262 - MT897458). The complete list of FAW population genome wide single nuclear polymorphic loci used is available from CSIRO’s public data access portal <https://data.csiro.au/collection/csiro:53315>, https://doi.org/10.25919/y3nd-2903.

Field-specific reporting

Please select the one below that is the best fit for your research. If you are not sure, read the appropriate sections before making your selection.

☐ Life sciences ☐ Behavioural & social sciences ☒ Ecological, evolutionary & environmental sciences

For a reference copy of the document with all sections, see nature.com/documents/nr-reporting-summary-flat.pdf

Ecological, evolutionary & environmental sciences study design

All studies must disclose on these points even when the disclosure is negative.

Study description	This study aims to test the hypothesis that the current global spread of the highly invasive noctuid moth pest Spodoptera frugiperda (fall armyworm, FAW) originated from an initial western African invasion in 2016 that spread across Africa to Asia and Southeast Asia. The study will employ reduced representation of genome-wide single nucleotide polymorphic (SNP) markers to examine genomic signatures of invasive and native populations to support/reject this hypothesis.
Research sample	The samples consisted of 17 populations of FAW from native North/Central/South American ranges (9 populations, 7 countries), and from invasive ranges (8 populations, 6 countries). The FAW were collected as larvae (mixed sex, predominantly 3rd-5th instars) from maize crops, or as adults (predominantly as males) via pheromone traps. Pre-border intercepted larval samples from Peru and China were also included. No manipulations were applied to collect the samples, and these were meant to represent populations under natural conditions for the purpose of whole genome sequencing to interrogate population genomic signatures and for molecular characterisation of known insecticide resistance genes, and the host-strain markers of mitochondrial genomes and the TPI partial gene located on the z-Chromosomes.
Sampling strategy	Larvae were collected directly from host plants and stored in ethanol until DNA extraction. Adults captured via pheromone traps were stored in ethanol until DNA extraction. No prior statistical methods were used to predetermine required sample size, as the high expected marker size through whole genome sequencing approach would compensate for lower biological sampling size.
Data collection	Genome data were generated from three independent research groups (France, China, Australia), and were collected and quality assessed by researchers responsible from those institutions prior to being up-loaded to the CSIRO's (Australia) server for analyses.
Timing and spatial scale	The invasive population range data collection was from 2016 in Western Africa, 2016/2017 Eastern Africa, 2018 (India), 2019 (China) and represented the initial populations that were reported in these countries. Native populations were sampled since 2009 until 2018, and represent existing endemic populations.
Data exclusions	Preserved Tanzanian FAW samples were significantly impacted by posting step to Australia, and only one individual was successfully sequenced. All remaining 9 individuals did not produce usable data and were excluded. No other samples were excluded.
Reproducibility	The population genomic approaches for the SNP markers have been described and published and are therefore highly reproducible. The methods and marker selection procedures have since been deployed in a follow-up study involving FAW populations from Southeast Asia (SEA), the Far East (FE), and Australia (Aust), and data generated from this study were successfully combined with the SEA/FE/Aust data to provide a global picture to highlight the Importance of multiple introductions in the invasive history of this pest.
Randomization	This is a survey of multiple populations - not a functional study, hence no randomisation is required. Standardised data generation and analysis was incorporated to allow for accurate analysis.
Blinding	Blinding was not relevant in the study as details of population country origins were needed to enable interpretation of genomic signatures.

Did the study involve field work? ☒ Yes ☐ No

Field work, collection and transport

Field conditions	varied (typically hot, humid)

Location	Field sites were in Florida, Mississippi, Puerto Rico, Guadeloupe, Mexico, French Guiana, Brazil, China, Malawi, Tanzania, Benin, Uganda, India. Peru (and one China) samples were from Australia's pre-border interception quarantine inspection services.
Access & import/export	Brazilian samples with export permit 18BR028445/DF was issued by IBAMA on 2018. Indian FAW gDNA were from Yainna et al. (2020; bioRxiv 2020.06.17.154880; doi: https://doi.org/10.1101/2020.06.17.154880) and the study of Sharanabasappa, et al. Pest Manag. Hortic. Ecosyst. 24, 23–29 (2018).
Disturbance	Minimal disturbance as specimens were pests of cultivated maize crop.

Reporting for specific materials, systems and methods

We require information from authors about some types of materials, experimental systems and methods used in many studies. Here, indicate whether each material, system or method listed is relevant to your study. If you are not sure if a list item applies to your research, read the appropriate section before selecting a response.

Materials & experimental systems

n/a	Involved in the study
☒	☐ Antibodies
☒	☐ Eukaryotic cell lines
☒	☐ Palaeontology and archaeology
☒	☐ Animals and other organisms
☒	☐ Human research participants
☒	☐ Clinical data
☒	☐ Dual use research of concern

Methods

n/a	Involved in the study
☒	☐ ChIP-seq
☒	☐ Flow cytometry
☒	☐ MRI-based neuroimaging
